# Cranial Migration of a Disseminated Thoracic Ependymoma Causing Transient Paraparesis: A Case Report

**DOI:** 10.1155/cro/8151806

**Published:** 2026-11-08

**Authors:** Kenta Kono, Tomonori Shigemura, Yohei Yamamoto, Miki Morimoto, Yasuaki Murata

**Affiliations:** ^1^ Department of Orthopaedic Surgery Teikyo University Chiba Medical Center Ichihara Chiba Japan teikyo-u.ac.jp

**Keywords:** case report, ependymoma, intradural extramedullary tumor, migration, paraparesis

## Abstract

We report a rare case of a disseminated ependymoma that caused acute neurological deterioration due to tumor migration after initial resection. A 56‐year‐old woman with a history of medullary ependymoma resection presented with progressive gait disturbance and thoracic pain. Magnetic resonance imaging (MRI) revealed multiple intradural extramedullary lesions along the thoracolumbar spine. After the first‐stage resection (T4–T10), she developed sudden lower limb weakness on postoperative day 11, and MRI demonstrated cranial migration of the residual tumor. The second‐stage resection (T11–L1) was performed, resulting in complete neurological recovery. Postoperative alterations in cerebrospinal fluid dynamics were considered a possible mechanism of tumor migration. This case highlights the potential for tumor movement in disseminated ependymoma and suggests that postoperative tumor migration should be considered when determining the surgical strategy.

## 1. Introduction

Several cases of migrating intradural extramedullary tumors have been reported, most of which are schwannomas [1–6]. Tumor migration has been attributed to elongation [4] or laxity [5, 7] of the nerve root, injection of intrathecal contrast medium during myelography [2, 8], postural changes [9], and Valsalva maneuvers [10]. Previous reports have mainly discussed diagnostic challenges, such as accurate localization of the tumor to prevent unnecessary laminectomy or repeated surgeries [7, 8]. Here, we describe a rare case of disseminated intradural extramedullary ependymoma that migrated cranially after initial resection, resulting in acute neurological deterioration. The aim of this report was to highlight the potential for postoperative tumor migration to cause acute neurological deterioration and to discuss the possible mechanisms underlying tumor migration and surgical considerations for patients with disseminated intradural extramedullary ependymoma.

## 2. Case Presentation

A 56‐year‐old woman presented with gait disturbance and pain radiating from the right back to the lateral thoracic region. Her symptoms had gradually worsened over 3 months, and she was referred to our department by a neurosurgeon at our institution. Ten years ago, she had undergone resection and radiation therapy for an ependymoma of the medulla oblongata at our hospital. At the time of the initial diagnosis, brain magnetic resonance imaging (MRI) was performed; however, MRI of the cervical, thoracic, and lumbosacral spine was not performed. Therefore, the presence or absence of spinal dissemination at the initial diagnosis could not be assessed. During the subsequent follow‐up period, brain MRI was performed regularly, approximately two to six times per year; however, spinal MRI surveillance was not performed.

Neurological examination revealed normal strength in both upper and lower limbs. The biceps, brachioradialis, and triceps reflexes were normal, whereas mild hyperreflexia was observed in the patellar and Achilles tendon reflexes. Sensory examination revealed mild hypoesthesia below the T11 dermatome. She exhibited spastic gait with instability. Routine laboratory tests showed no remarkable abnormalities. Spinal MRI was then performed and demonstrated multiple intradural extramedullary tumors distributed along the thoracic and lumbar spinal canal (Figure 1a,b). Based on these findings and her history of intracranial ependymoma, dissemination of ependymoma to the spinal intradural extramedullary space was suspected. Because the lesions were widespread, a two‐stage surgery was planned to avoid the anticipated surgical burden of attempting complete resection in a single procedure. The first stage consisted of resection and fixation from T4 to T10, followed by tumor removal from T11 to L1 in the second stage.

**Figure 1:**
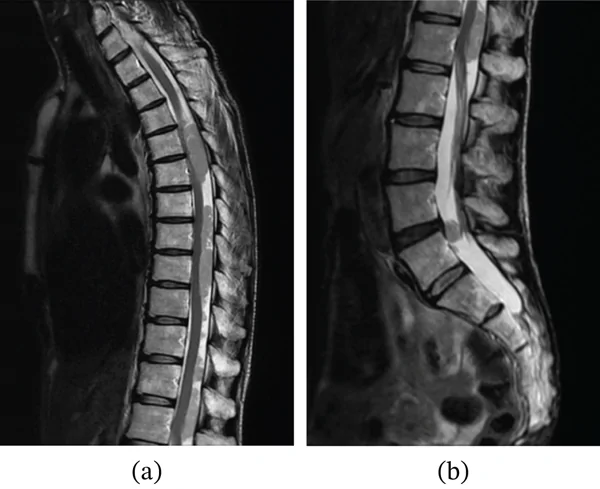
Preoperative MRI of the thoracolumbar spine. T2‐weighted MRI showing multiple intradural extramedullary tumors along the thoracolumbar spine. (a) Thoracic spine. (b) Lumbar spine.

During the first surgery, laminectomies and posterior fusion were performed from T4 to T10, followed by tumor resection at the same levels. Histopathological examination revealed an ependymoma. The tumor showed papillary and tubular proliferation around fibrovascular stromal cores with characteristic perivascular pseudorosettes (moose track pattern). Although cellularity was relatively high, mitotic figures were inconspicuous and no regional necrosis was identified. The MIB‐1 labeling index was approximately 7%. The findings were consistent with a WHO Grade II ependymoma based on the fourth Edition of the WHO Classification of Tumors of the Central Nervous System. The dura mater was closed with sutures and further reinforced using Beriplast P Combi‐Set (fibrin sealant containing fibrinogen and factor XIII; CSL Behring K.K., Tokyo, Japan) and Neoveil Sheet (absorbable polyglycolic acid [PGA] sheet for tissue reinforcement; Gunze Medical Limited, Osaka, Japan). Finally, posterior fixation from T4 to T10 was performed. Motor evoked potential (MEP) monitoring remained stable throughout the surgery. The total blood loss was 720 mL, and the surgical time was 7 h and 14 min. No immediate postoperative weakness was observed. However, on postoperative day 1, mild right lower limb weakness was observed, with the iliopsoas muscle strength graded as MMT 3 on the right and MMT 4 on the left. Initially, spinal cord compression due to a postoperative hematoma was suspected, and an MRI scan was performed. However, MRI showed spinal cord edema, and no hematoma was detected. There was no radiological evidence of tumor migration at that time. On postoperative day 11, the patient suddenly developed severe weakness of the right lower limb, with the right tibialis anterior muscle strength graded as MMT 1. MRI was performed again and revealed cranial migration of the residual tumor. The lesion, which had been located at the superior aspect of the T12 vertebral body before surgery (Figure 2a), had migrated cranially beyond the inferior endplate of T11 (Figure 2b).

**Figure 2:**
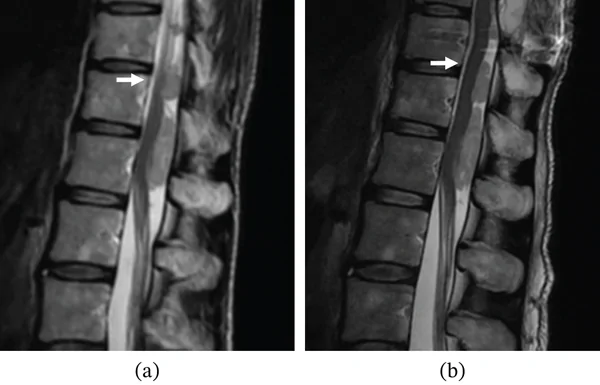
Cranial migration of the residual tumor after the first‐stage operation. MRI showing the positional change of the residual intradural extramedullary tumor. (a) Preoperative MRI showing the tumor at the superior aspect of the T12 vertebral body (arrow). (b) MRI on postoperative day 11 showing cranial migration of the residual tumor (arrow).

The second planned operation was performed 2 weeks after the first surgery, involving tumor resection following laminectomies from T11 to L1. The dura mater was carefully sutured and reinforced using the same method as in the first surgery. The total blood loss was 320 mL, and the surgical time was 3 h and 44 min. Histopathological examination of the second‐stage specimen showed findings similar to those of the first‐stage specimen, including papillary proliferation around fibrovascular stromal cores and a characteristic perivascular pseudorosette (moose track pattern). No obvious necrosis, marked nuclear atypia, or mitotic figures were identified. The MIB‐1 labeling index was approximately 7%–8%. During the procedure, the MEP waveform of the tibialis anterior muscle reappeared (Figure 3a,b).

**Figure 3:**
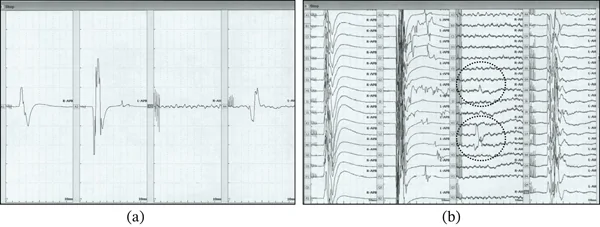
Changes in motor evoked potentials during the second‐stage operation. (a) Before the second‐stage operation, no MEP waveform was detected from the tibialis anterior muscle. (b) During tumor resection, the MEP waveform reappeared (dotted circle).

After the second surgery, the patient was followed clinically and with MRI for 9 months. At the 9‐month follow‐up, she had regained full muscle strength. She subsequently did not attend scheduled follow‐up visits. Approximately 4 years after surgery, MRI of the thoracolumbar spine was performed for another clinical indication and showed no evidence of tumor recurrence. The clinical course and treatment timeline are summarized in Figure 4.

**Figure 4:**
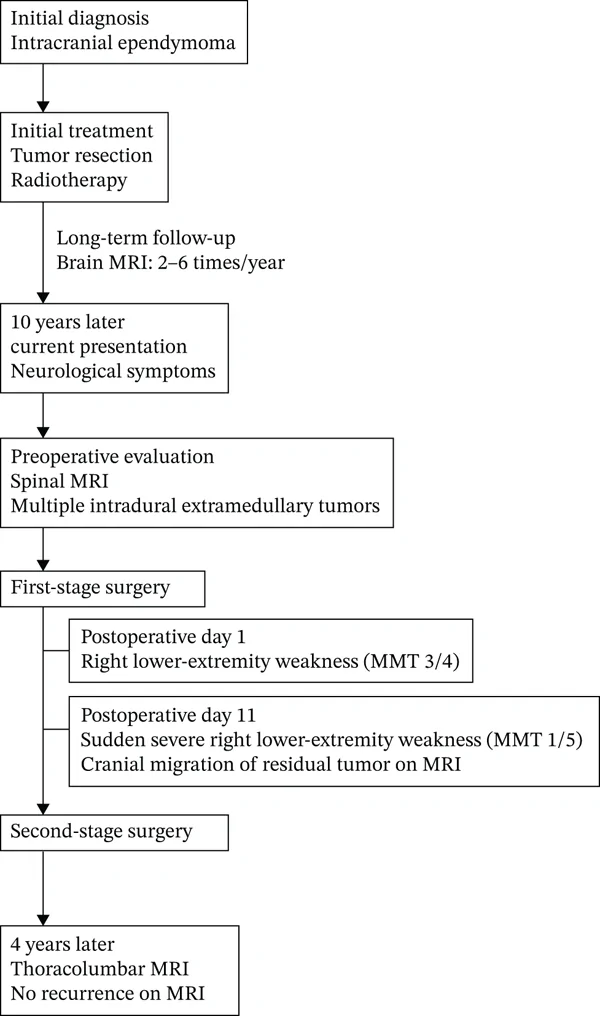
Timeline of the patient′s clinical course. The timeline summarizes the major clinical events and treatment course.

## 3. Discussion

We experienced a rare case of disseminated intradural tumor in which progressive paraparesis occurred because of cranial migration of the residual tumor after the first of two planned surgeries.

Possible mechanisms of tumor migration include nerve root elongation due to tumor weight [4], nerve root laxity [5, 7], contrast injection during myelography [2, 8], postural changes [9], and Valsalva maneuvers [10]. In the present case, postoperative CSF leakage was considered a possible mechanism contributing to tumor migration. The postoperative drain contained serous fluid and was maintained for 7 days after surgery. Given that the dura had been opened during the intradural procedure, postoperative CSF leakage was suspected; however, no definitive imaging evidence of CSF leakage was available. Therefore, a causal relationship between CSF leakage and tumor migration cannot be established. Nevertheless, postoperative alterations in CSF dynamics may have contributed to cranial displacement of the residual tumor. A reduction in pressure at the cranial surgical site could potentially create a relative pressure gradient within the subarachnoid space. In the presence of loosely attached disseminated intradural tumor nodules, such alterations in CSF dynamics may facilitate cranial displacement.

The decision to perform the surgery in two stages was based on the extensive distribution of the intradural tumors and the anticipated surgical burden of attempting complete resection in a single procedure. Although a single‐stage procedure might theoretically reduce the opportunity for postoperative tumor migration of residual lesions, the present case cannot establish that single‐stage resection is superior to staged surgery. The potential risk of postoperative tumor migration should therefore be considered when determining the surgical strategy, whereas the choice between single‐stage and staged resection should be individualized according to tumor extent and anticipated surgical risks.

This case has several diagnostic limitations. First, spinal MRI was not performed at the time of the initial diagnosis or during the intervening 10 years. Therefore, it is not possible to determine whether the spinal lesions represented delayed dissemination or previously undetected asymptomatic disease. Second, molecular genetic testing, including MYCN amplification analysis, was not performed. Thus, the molecular classification of the spinal ependymoma remains uncertain.

## 4. Conclusion

We report a case of transient neurological deterioration caused by cranial migration of a disseminated intradural extramedullary ependymoma after initial thoracic resection. Postoperative alterations in CSF dynamics were considered a possible mechanism underlying tumor migration. Although the patient achieved full neurological recovery, this case highlights the potential risk of tumor migration following staged resection. The potential risk of postoperative tumor migration should be considered when determining the surgical strategy, whereas the choice between single‐stage and staged resection should be individualized according to tumor extent and anticipated surgical risks.

## Funding

No funding was received for this manuscript.

## Ethics Statement

According to the institutional policies of Teikyo University, ethical approval was not required for this case report.

## Consent

Written informed consent was obtained from the patient for publication of this case report and the accompanying images.

## Conflicts of Interest

The authors have nothing to report.

## Data Availability

Research data are not shared.
